# Prevalence and profiles of unmet healthcare need in Thailand

**DOI:** 10.1186/1471-2458-12-923

**Published:** 2012-10-30

**Authors:** Noppakun Thammatacharee, Kanjana Tisayaticom, Rapeepong Suphanchaimat, Supon Limwattananon, Weerasak Putthasri, Rajana Netsaengtip, Viroj Tangcharoensathien

**Affiliations:** 1 International Health Policy Program, Ministry of Public Health, Nonthaburi, Thailand; 2 Faculty of Pharmaceutical Sciences, Khon Kaen University, Khon Kaen, Thailand; 3 National Statistical Office, Bangkok, Thailand

**Keywords:** Unmet need, Inequity, Access to healthcare, Health policies

## Abstract

**Background:**

In the light of the universal healthcare coverage that was achieved in Thailand in 2002, policy makers have raised concerns about whether there is still unmet need within the population. Our objectives were to assess the annual prevalence, characteristics and reasons for unmet healthcare need in the Thai population in 2010 and to compare our findings with relevant international literature.

**Methods:**

A standard set of OECD unmet need questionnaires was used in a nationally-representative household survey conducted in 2010 by the National Statistical Office. The prevalence of unmet need among respondents with various socio-economic characteristics was estimated to determine an inequity in the unmet need and the reasons behind it.

**Results:**

The annual prevalence of unmet need for outpatient and inpatient services in 2010 was 1.4% and 0.4%, respectively. Despite this low prevalence, there are inequities with relatively higher proportion of the unmet need among Universal Coverage Scheme members, and the poor and rural populations. There was less unmet need due to cost than there was due to geographical barriers. The prevalence of unmet need due to cost and geographical barriers among the richest and poorest quintiles were comparable to those of selected OECD countries. The geographical extension of healthcare infrastructure and of the distribution of health workers is a major contributing factor to the low prevalence of unmet need.

**Conclusions:**

The low prevalence of unmet need for both outpatient and inpatient services is a result of the availability of well-functioning health services at the most peripheral level, and of the comprehensive benefit package offered free of charge by all health insurance schemes. This assessment prompts a need for regular monitoring of unmet need in nationally-representative household surveys.

## Background

It was not until 2002 that the entire population of Thailand was fully covered by financial risk protection schemes. At present, government employees and their dependents are covered by the tax-financed Civil Servant Medical Benefit Scheme (CSMBS), private sector employees by the payroll-tax financed Social Health Insurance (SHI) scheme, and the remaining population by the tax-financed Universal Coverage Scheme (UCS) [1].

A comprehensive benefit package is offered by all three insurance schemes. The package covers all outpatient and inpatient services including medicines listed in by the National Lists of Essential Medicines, high cost care, accident and emergency services, and health promotion and preventive services. All of the schemes are subject to a small exclusion list, which includes items such as non-clinically indicated cosmetic surgery. For SHI members, all services are provided free at contractor provider where members registered with. Patients who bypass the contractor providers without proper referral are liable to pay in full. Similarly, UCS members are required to register with a contractor provider network, from which services are provided at no cost. Patients bypassing the registered providers are liable to pay in full [1]. There is no registration requirement for CSMBS members.

Empirical evidence shows a significant increase in healthcare use for both outpatient and inpatient services following the introduction of the UCS [2]. In that study, the use of health services was pro-poor [2]. Financial risk protection for all has been achieved by UCS, resulting in a minimal prevalence of catastrophic health expenditure and financial hardship due to health payment—so-called health impoverishment [3,4]. Government budget subsidies preferentially benefit the poorer members of the UCS [5]. The household out-of-pocket payment for health reduced from 33% of total health expenditure in 2001 (prior to universal coverage) to 18% in 2008 [6]. This level of out-of-pocket payment is on par with an OECD average of 19.8% in 2009 [7].

On its own, financial risk protection offered by a comprehensive set of service packages provided free at point of service, as practised by all three insurance schemes, does not necessarily resolve other barriers of access to and use of healthcare. For example, when distant services are available, patients may not be able to afford the travel costs, or regular transport may not be available. Where services are available, other problems might exist. For example, health workers are not responsive to patients’ needs, service hours not suit the patients, and staffs may have poor attitudes or may not observe confidentiality. Other socio-cultural factors such as low literacy and a lack of awareness of service availability also play critical roles. Literature shows that interactions between supply and demand characteristics determine actual use [8]. Medical care use is also influenced by patient perceptions of illnesses severity [9]. It has been documented that cancer patients experience a high level of unmet need [10].

Even in the context of universal coverage, unmet need may exist when there are other barriers to seeking the necessary healthcare [11], resulting in increased morbidity and healthcare costs [12]. Unmet need has been assessed among various vulnerable and disadvantaged population groups, e.g. minority ethnic groups, the uninsured population and children [13-17], and among patients suffering from specific diseases, such as mental illness [18] cancer [19] or HIV, for which social stigmatization is a major barrier [20]. Women in British Columbia, Canada are more likely to have high levels of unmet need because of their roles in the family hindering them from getting the necessary health care [21]. Another study found that children and adolescents from lower income families are more likely to report having unmet need [22].

These studies provide a better understanding of unmet health need among patients with specific diseases or sub-population groups, as well as the reasons for not seeking care and the factors influencing unmet health need. However, they do not provide regular, nationally-representative data from which policies can be devised and mitigate. To address this gap, regular monitoring of unmet need is conducted through household surveys in OECD countries. It is noted that routine comparable data are collected in most OECD countries. These include the indicators of inequalities in self-rated health, self-rated disability, the extent of public and private health care coverage, and self-reported unmet medical and dental care need [23].

National data from the OECD countries has demonstrated significant levels of unmet need among the poor and people with physical limitations on daily activities; in contrast, the elderly, men and lower educated groups tend to report less unmet need [24]. High rates of unmet need for medical examinations among the adult population were reported in Poland (9.3%), Greece (5.8%) and Portugal (5.0%) in 2006. However, inequalities within countries were greater in countries such as Belgium, Portugal and the Slovak Republic, although in Belgium the overall reported level of unmet need was low (less than 1%).

The Health and Welfare Survey (HWS), equivalent to the Demographic and Health Survey, has been regularly conducted in Thailand by the National Statistical Office (NSO). It was conducted every five years prior to the achievement of universal health coverage in 2002, following that, it was conducted over five consecutive years (2003–2007) to monitor the impact of the universal coverage scheme on households, and thereafter every two years (2009, 2011). Unfortunately, the HWS did not include an assessment of unmet need. To respond to policy concerns about potential unmet need in the context of universal coverage, this study assessed the magnitude, profiles and reasons for unmet need among the Thai population in 2010 and compared our findings with those of relevant international studies.

## Methods

### Data source

In response to evidence based policy formulation, guiding implementation and supporting monitoring and evaluation at household level, a Panel Socio-Economic Survey (Panel SES) was initiated by the NSO in 2005 with the aim of maintaining a long-term panel of households. The first three waves were conducted in 2005, 2006 and 2007 which covered 6,000 nationally-representative households. Interview was conducted in Thai language by NSO field staffs for which each member in sample households was face-to-face interviewed. For person who were absent more than three times and children aged under 14, head of households would answer the questionnaire. However, proxy respondents are not allowed for health modules. Comparing to the number of households in the first wave, the NSO was able to retain samples of 96.2% (5774 out of the 6000 original households) in the second wave, 93.1% (5584 households) in third wave and 91.2% (5469 households) in the fourth wave. The sample was selected using stratified two-stage sampling covering all 76 provinces of Thailand, broken down by municipal and non-municipal areas. The survey contained detailed questions on socio-economic status (income, expenditure, ownership of durable goods and household characteristics), self-reported illness and use of healthcare services.

In the European Union Statistics on Income and Living Conditions (EU-SILC) survey, a set of standard questions on unmet need is asked:

Was there any time during the last 12 months when you personally, really needed a medical examination or treatment for a health problem but you did not receive it? Yes/No

What was the main reason for not consulting a medical specialist? Could not afford to (too expensive)/Waiting list/Could not take time off work (or could not take time off from caring for children or others)/Too far to travel or no means of transport/Fear of doctor, examination, treatment/Wanted to wait and see if problem got better on its own/Didn’t know any good doctor or specialist/Other reason.

Using the EU-SILC survey as a basis, a standard set of questions on unmet need was added in the fourth wave of the Panel SES in 2010 to assess the prevalence and profile of unmet need across respondents with different socio-economic characteristics. This is the first attempt to assess unmet healthcare need in Thailand. It is planned to maintain prospective monitoring of unmet healthcare need in Thailand in the future.

### Analytical methods

We estimated the annual prevalence of unmet health need by socio-economic characteristics of the population and identify the reasons for the unmet health need, and compared results with selected OECD countries. The un-weighted 21,770 samples were extrapolated to 63.87 million total Thai population, using sampling weights provided by NSO. All variables are expressed as percentages and chi-square statistics were used to determine statistical significant differences. A Principal Component Analysis [25] was applied to generate an asset index value for each household. All households were classified by the asset quintiles, the first quintile is the poorest 20%, and the fifth quintile is the richest 20% of population.

## Results

### Sample profiles

Table 1 presents the socio-economic characteristics of the study population. The insurance coverage, gender, age and geographical distributions adequately represent the Thai population.

**Table 1:** Population characteristics

**Characteristic**	Survey population*	Thai population**
	**% (95% CI)**	**%**
Male	48.7 (48.4-49.0)	49.2
Female	51.3 (51.0-51.6)	50.8
Age		
0–4	6.8 (6.7-6.9)	6.2
5–14	14.6 (14.4-14.8)	13.7
15–59	66.8 (66.5-67.1)	68.4
>60	11.9 (11.7-12.0)	11.7
Domicile region		
Bangkok and vicinity	15.6 (14.8 -16.3)	14.0
Central	19.7 (19.5-19.9)	19.8
North	18.5 (18.3-18.7)	18.5
Northeast	32.0 (31.7-32.3)	33.8
South	14.2 (14.0-14.4)	13.9
Area		
urban	32.9 (32.6-33.2)	N/A
rural	67.1 (66.8-67.4)	
Insurance coverage		
UCS	76.4 (76.2-76.6)	N/A
SHI	12.5 (12.3-12.6)	
CSMBS	11.1 (11.0-11.2)	
Total population, million	63.9	63.7

Note: * Survey population data is extrapolated to the Thai population.

**Thai population 2010, the Ministry of Interior.

### Inequity in the prevalence of unmet need

The annual prevalence of unmet outpatient and inpatient healthcare need was broken down by various socio-economic strata, see Table 2.

**Table 2:** Annual prevalence and socio-economic profiles of unmet need for outpatient and inpatient services, 2010

**National prevalence, %**	**Outpatient services**	**Inpatient services**
Overall	1.4	0.4
By area		
Urban	1.1	0.6
Rural	1.6	0.3
By gender		
Female	1.6	0.5
Male	1.3	0.3
By age group		
Under 5 years	0.3	0.2
5–14 years	0.5	0.1
15–59 years	1.6	0.4
>60 years	2.4	0.7
By geographical region		
Grater Bangkok	1.0	0.4
Central	1.4	0.4
North	1.7	0.5
Northeast	1.7	0.3
South	0.9	0.7
By insurance coverage		
UCS	1.6	0.5
SHI	1.0	0.2
CSMBS	0.9	0.3
By asset index quintile		
Q1 (poorest)	2.6	0.7
Q2	1.3	0.4
Q3	1.4	0.3
Q4	0.7	0.3
Q5 (richest)	1.1	0.3

Note: p < 0.001 for all comparison categories, sample weight applied.

A common trend emerged. The prevalence of unmet need for ambulatory care (1.4%), although small, was significantly (p < 0.001) higher than the prevalence of unmet need for hospitalization (0.4%) both at the national level and in a subgroup analysis. Females had a higher level of unmet need for both services than males. There was an age gradient of unmet need for both services--increased need with age. The rural population reported higher levels of unmet need for ambulatory services, but lower unmet need for hospital admission compared with the urban counterparts. Unmet need was lowest for both services in the Greater Bangkok region where there is a greater supply of healthcare services than there is in other regions. The poorer North and Northeast regions had the highest level of unmet need for outpatient services, whereas the South region had the highest level of unmet need for hospital admission. Concerning economic status measured as the asset index, the poorer people were, the higher their unmet need for both services. The rich-poor gap was more than double. Members covered by CSMBS reported a lower level of unmet need than those covered by UCS.

### Causes of unmet need

Despite the low prevalence of unmet need, the reasons for outpatient unmet need varied (Table 3). Two amenable factors were identified: first, that it was too far for respondents to travel to a health care provider, and second, that they could not afford the treatment. Other reasons were related to socio-cultural dimensions of health seeking behaviour, such as not being aware of effective treatment, poor impressions of providers, being unable to take time off to seek care and no companion to services.

**Table 3:** Reasons for not using services among those reporting unmet need for outpatient care, number of sample and % (in parenthesis)

**Reasons**	**National**	**Gender**		**Age**		**Area**		**Region**		**Asset index**		**Insurance**	
		**Male**	**Female**	**5-14 year**	**> 60 year**	**Urban**	**Rural**	**BKK**	**NE**	**Q1**	**Q5**	**CSMBS**	**UCS**
Too far to travel	45	23	22	2	25	10	35	6	24	16	4	2	43
	(14)	(20)	(9)	(10)	(34)	(12)	(14)	(24)	(22)	(21)	(4)	(6)	(15)
Cannot afford treatment	5	2	3	0	2	1	4	1	3	1	0	0	5
	(1)	(2)	(1)	(0)	(1)	(0)	(2)	(1)	(3)	(0)	(0)	(0)	(2)
Not sure there are effective treatments	40	19	21	0	21	8	32	7	11	9	8	1	37
	(16)	(18)	(15)	(0)	(22)	(5)	(20)	(9)	(15)	(13)	(26)	(3)	(18)
Bad impression of providers	19	7	12	2	6	5	14	5	6	2	5	2	15
	(5)	(2)	(7)	(9)	(4)	(3)	(6)	(14)	(3)	(1)	(8)	(6)	(3)
No time to seek care	60	27	33	1	9	25	35	10	19	16	7	5	44
	(24)	(25)	(24)	(2)	(7)	(29)	(23)	(18)	(27)	(31)	(24)	(16)	(22)
No accompanies	21	9	12	0	14	8	13	4	10	5	2	1	20
	(4)	(4)	(3)	(0)	(11)	(4)	(3)	(3)	(5)	(2)	(3)	(2)	(4)
Cannot afford transportation	7	3	4	1	2	2	5	1	2	2	0	0	7
	(1)	(1)	(1)	(4)	(1)	(2)	(1)	(3)	(1)	(1)	(0)	(0)	(2)
Other reasons	112	51	61	7	25	42	5	15	26	25	17	10	96
	(34)	(28)	(39)	(74)	(20)	(46)	(31)	(29)	(24)	(30)	(36)	(67)	(34)
Total %	309	141	168	13	104	101	70	49	101	76	43	21	267
	(100)	(100)	(100)	(100)	(100)	(100)	(100)	(100)	(100)	(100)	(100)	(100)	(100)

Notes:

1. The raw numbers of samples are provided, the percentage in parenthesis was calculated from weighted population representing the national figures.

2. p < 0.001 for all comparison categories, sample weight applied for the percentage in parenthesis.

3. BKK: Greater Bangkok, NE: Northeast region.

Resulting from the comprehensive benefit package offered by all three health insurance schemes and zero co-payment for services, only a small proportion of respondents who have unmet need (1%) gave “cannot afford to pay for treatment” as their reason. However, a slightly higher proportion of respondents who have unmet need (3%) in the Northeast region said they were unable to pay for health services.

The geographical barrier remains a problem when 14% of the unmet need respondents voiced the opinion that health services are too far away to access. This opinion was particularly prevalent among those in the poorest quintiles (21%) and the UCS members (15%). The geographical barrier is amendable by extending health services closer to the respondent’s domicile. The most common reason for having unmet need was not being able to take time off to seek outpatient care (reported by 24% among the unmet need respondents), particularly among the rural population living in the Northeast region, and the poorest quintile and UCS members. In addition, it was not uncommon for respondents to doubt whether effective treatment is available, this was reported by 16% of those with unmet need.

Reasons for having unmet need for inpatient services were different from those for outpatient services (Table 4). They were both reported by 17% of the respondents with unmet need whereas the affordability of travel cost was reported zero. Notably, more respondents who were UCS members (20%), from rural areas (27%), the Northeast region (57%) or the poorest quintile (13%) reported geographical barriers to seeking health care than respondents who were members of CSMBS, from urban areas, Bangkok or the richest quintile. Financial difficulties in paying for treatment (reported by 17% of all respondents) were more commonly reported among the rural population (21%), poorest quintiles (31%), and UCS members (19%) than among their comparative groups. However, ‘no time to seek care’ was equally important (17%), and more common among men, those living in urban areas or Bangkok, and CSMBS members than among their comparative groups. It is noticeable that the overall percentage of ‘other reasons’ was quite high for both outpatient and inpatient services, 34% and 31% respectively.

**Table 4:** Reasons for not using services among those reporting unmet need for inpatient care, number of samples and % (in parenthesis)

**Reasons**	**National**	**Gender**		**Age**		**Area**		**Region**		**Asset index**		**Insurance**	
		**Male**	**Female**	**5-14 year**	**> 60 year**	**Urban**	**Rural**	**BKK**	**NE**	**Q1**	**Q5**	**CSMBS**	**UCS**
Too far to travel	16	5	11	1	3	5	11	1	6	7	0	0	16
	(17)	(8)	(22)	(49)	(9)	(6)	(27)	(5)	(57)	(13)	(0)	(0)	(20)
Cannot afford treatment	5	2	3	1	0	3	2	1	1	2	1	0	5
	(17)	(14)	(18)	(30)	(0)	(12)	(21)	(4)	(3)	(31)	(4)	(0)	(19)
Not sure there are effective treatments	4	1	3	0	1	1	3	0	2	1	0	0	4
	(6)	(11)	(3)	(0)	(3)	(8)	(3)	(0)	(5)	(2)	(0)	(0)	(6)
Bad impression of providers	3	0	3	0	1	0	3	0	1	2	0	0	3
	(2)	(0)	(3)	(0)	(5)	(0)	(4)	(0)	(5)	(5)	(0)	(0)	(3)
No time to seek care	13	6	7	1	5	9	4	2	5	0	2	2	10
	(17)	(23)	(14)	(21)	(16)	(31)	(5)	(23)	(20)	(0)	(24)	(38)	(11)
No accompanies	4	2	2	0	3	2	2	1	2	1	1	0	4
	(10)	(4)	(12)	(0)	(42)	(19)	(1)	(57)	(7)	(22)	(6)	(0)	(11)
Cannot afford transportation	0	0	0	0	0	0	0	0	0	0	0	0	0
	(0)	(0)	(0)	(0)	(0)	(0)	(0)	(0)	(0)	(0)	(0)	(0)	(0)
Other reasons	27	13	14	0	10	8	19	2	1	8	3	3	18
	(31)	(39)	(27)	(0)	(26)	(23)	(38)	(10)	(3)	(28)	(66)	(62)	(29)
Total %	72	29	43	3	23	28	44	7	18	21	7	5	60
	(100)	(100)	(100)	(100)	(100)	(100)	(100)	(100)	(100)	(100)	(100)	(100)	(100)

Notes

1. The raw numbers of samples are provided, the percentage in parenthesis was calculated from weighted population representing the national figures.

2. p < 0.001 for all comparison categories, sample weight applied for the percentage in parenthesis.

3. BKK: Greater Bangkok, NE: Northeast region.

### Thailand unmet health needs in the international context

**Figure 1:**
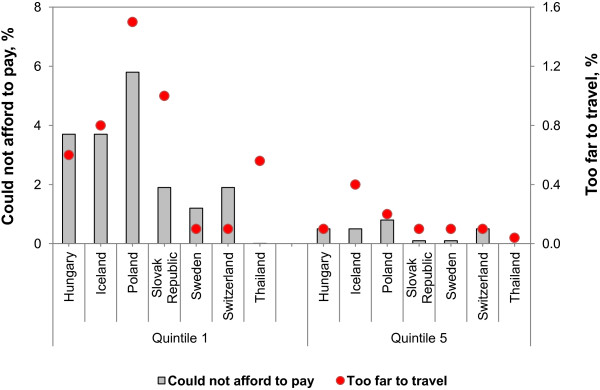
**Unmet need for outpatient services due to cost and geographical barrier.** 1. OECD refer to Unmet need for a medical examination, selected reasons by income quintile, European countries, 2009. 2. Thailand data from Panel SES 2010.

Figure 1 compares the prevalence of unmet need for outpatient services due to cost and geographical barriers, comparing the poorest and richest quintiles in Thailand and selected OECD countries where comparable data allows for unmet need due to both healthcare cost and geographical barriers [26]. Thailand performs well in financial risk protection, for both poorest and richest quintiles, with only 0.01% of the poorest quintile reporting unmet need because they could not afford it. In quintile 5 the corresponding percentage was 0.

However, of those poorest quintile in Thailand, 0.56% had unmet need due to geographical barriers, which is comparable to reports from Hungary and Iceland but lower than those from Poland and the Slovak Republic. Among the richest quintiles, 0.04% report having unmet need due to geographical barriers, which is much lower than the reports from Iceland but comparable with others.

## Discussion

The identification numbers of primary sampling unit and the strata were not available in this dataset for which an analysis using the complex survey design is not possible. The sampling weights were applied instead. As such, the point estimate will not be biased even though the standard error will be slightly different from the analysis using complex design. Also, the survey sample size is large, more than 6,000 households, the standard error should not be too large which compromises the statistical significance level.

Two amendable factors contribute to the low prevalence of unmet need: the adequate distribution of healthcare services and the comprehensive benefit package provided free of charge to UCS, CSMBS and SHI members.

**Figure 2:**
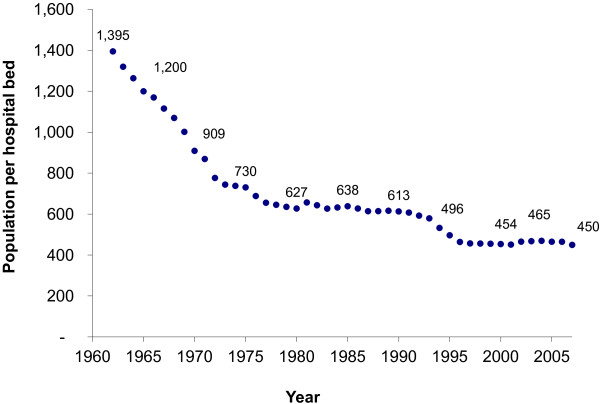
Population per hospital bed, 1960–2007.

The geographical coverage of healthcare services, which has extended to the most peripheral level of district and sub-district, is reflected in Figure 2. The hospital bed-population ratio reduced from 1:1395 in 1960 to 1:450 in 2007. This is a result of two decades of significant government investment in district hospitals between 1960 and 1980. In addition to the expansion of the geographical distribution and number of hospitals, the government invested in health centres to provide primary healthcare services to the rural population at the sub-district level. Health centre coverage improved from a ratio of 1:29,700 in 1960, to 1:6400 in 2007. Health centres and district hospitals are “close-to-client” service providers, and are easily accessed by the population because of their geographical proximity. They are major contributors to the low prevalence of unmet need due to geographical barriers.

In addition to infrastructure, the government has invested in the training of health workers, notably doctors and nurses. Various policy instruments were introduced to retain them in rural health services [27], such as government bonding for mandatory services by all new health-related graduates, financial and non-financial incentives, and other interventions recommended by WHO [28]. The doctor-population ratio reduced from 1:10,100 in 1960 to 1:2800 in 2007. In addition, the nurse-population ratio reduced from 1:5300 in 1960 to 1:500 in 2007. Significant improvements in the doctor/nurse-population ratios were associated with other policy mechanisms requiring service in rural areas, although an inequitable distribution across geographical regions remains [29].

The extension of financial risk protection minimized the prevalence of unmet need due to cost. Comprehensive benefit packages, including coverage for outpatient and inpatient services, medicines, diagnostic and high cost care such as cancer therapy and heart surgery, and point of service costs are major determinants of the low prevalence of unmet need due to cost. This has also been documented elsewhere [1,4,30,31].

Despite the low prevalence of unmet need, there are still inequities against women, rural residents, the Northeast region, UCS members and the poorest quintile. Policy makers are well aware of the affluence benefit and inefficiency in the CSMBS due to provider payment methods, and policy efforts to harmonize the three insurance schemes are underway. In addition, although all three insurance schemes offer a comprehensive benefit package, some members chose not to use and bypassed the free health services entitled to them and paid in full. Also services such as self-prescribed medicines in private pharmacies and private clinics were not covered by insurance.

One limitation of the survey revealing the reasons for unmet health need is that only the primary reason was allowed for the respondents. As such, the less important reasons would be under reported.

## Conclusions

A number of factors explain the low prevalence of unmet need. The comprehensive benefit package and free treatment at point of service provide financial risk protection to UCS members—minimizing unmet need due to cost. The extensive geographical coverage of health services and district health services minimized the geographical barriers to seeking heath care. These “close-to-client services” are easily accessible because of their geographical proximity to their target population.

Useful evidence generated from this assessment prompts the policy recommendation that unmet need should be monitored on a regular basis. The good institutional relationship between health and statistical constituencies supports the regular monitoring of unmet need in the biannual Health and Welfare Survey.

## Competing interests

None to declare.

## Authors' contributions

All co-authors contributed to the conceptualization of the paper. RN collected data. KT and SL analyzed the data. VT, NT, RS and WP drafted the manuscript. All authors contributed to the shaping of the manuscript and approved the final version.

## Pre-publication history

The pre-publication history for this paper can be accessed here:

http://www.biomedcentral.com/1471-2458/12/923/prepub
